# Editorial Notes

**Published:** 1894-11

**Authors:** 


### Editorial Notes.

---

Dr. Howard A. Kelly, has now moved to 1406 Eutaw Place, Baltimore, Md.

---

Football Casualties.—*The Lancet* of October 20th, chronicles three deaths and four fractures as the result of the previous week's sport.

---

The bronze statue erected by the friends of the late Dr. J. Marion Sims was unveiled Saturday afternoon in Bryant Park. Dr. George M. Shrady and Dr. Paul F. Munde made addresses, and Park Commissioner Tappen accepted the statue on behalf of the city.

---

The seventh annual meeting of the Southern Surgical and Gynecological Association is in session in Charleston, S. C., during the present week, Nov. 13, 14 and 15th. We hope to present our readers with several interesting papers to be read at this meeting.

---

Dr. M. G. Campbell, late house surgeon to the Grady Hospital, has decided to locate in this city. Dr. Campbell graduated at the Atlanta Medical College, class '94, taking first honor. We extend him a cordial welcome and predict much success to our genial friend.

---

Sanders & Son's Eucalypton Extract (Eucalyptol).—Whenever mention is made of "Oil of Eucalyptus," we beg you to bear in mind that such reference applies to our preparation, styled for distinction, "Eucalypti Extract (Eucalyptol)." To avoid disappointment, we would suggest to specify, when prescribing, our manufacture. Samples gratis through Dr. Sanders, Dillon, Iowa. Meyer Bros. Drug Co., St. Louis, Mo.

WANT NO PHYSIOLOGY.—The Christian Scientists of Burlington, Ia., have petitioned the school board to excuse their children from attendance when physiology is taught. The petitioners declare that there is no material body, and object to having their children taught to believe that there is anything so much in evidence as a stomach or a liver.—*Times and Register.*

---

DR. WILLIAM GOODELL, the eminent gynecologist, died at his residence in Philadelphia, on Oct. 27th, aged sixty-five years. In 1865, Dr. Goodell was appointed physician-in-chief of the Preston Retreat, for diseases of women, and held this position until his health failed. He was also professor of gynecology in the University of Pennsylvania until two years ago.

---

HONOR TO A NEW YORK SURGEON.—Dr. Arpad G. Gerster has received from the Emperor of Austria and King of Hungary, the high distinction of the Knight's Cross of the Order of Francis Joseph, in grateful recognition of his valuable philanthropic labors in founding the Hungarian Emigrant Aid Association of this city. This is the first instance in which an American citizen has received this honor who has not held office under the Austrian government. It is eminently fitting also that a member of our profession should be thus closely associated with such noble work, and that he should be so worthily rewarded.

---

A GENTLE LAXATIVE.—The profession as well as the public have long appreciated the importance of a simple laxative. Time out of mind remedies have been in every-day use in the home for this purpose, but it remained for the California Fig Syrup Company to furnish a pleasant, potent, perfect laxative, safe to be used in the home of members of the family of all ages.

The Company has frankly informed the medical profession that the chief laxative ingredient of their compound is senna, so treated that all tendency on its part to gripe and produce irritation and subsequent debility in the bowels, is removed. The chief feature claimed by the Company for their Syrup of Figs is the fact that the component parts of the product have all disagreeable taste disguised by a mingling of aromatic carminatives in such a way as to make it really pleasant to the taste; and these aromatics at the same time overcome all disposition upon the part of the drug to pain and discomfort; and carrying as it does the stamp of the Company's responsibility, it is always reliable and uniform in its effects.

It is conceded by every practical physician that a family laxative is one of the few medicinal agents which they will entrust to family use, and surely anything which will tend to assist in the relief of that *bete noire* of child and adult life—constipation, is a helper in the direction of general healthfulness.

The medical profession has not only consented to the use upon the part of the families under their care of Syrup of Figs, but when desiring to order gentle purgatives and simple laxatives they cheerfully specify in their prescriptions the product referred to; and the wonderful success of this gentle family laxative is largely owing to its universal use by the medical profession.—*Medical Mirror.*

---

CHICAGO GYNECOLOGICAL SOCIETY.—At the sixteenth annual meeting of the Chicago Gynecological Society, held Oct. 19, 1894, the following officers were elected to serve the ensuing year: Dr. Franklin H. Martin, President; Dr. A. J. Foster, First Vice-President; Dr. J. C. Hoag, Second Vice-President; Dr. H. P. Newman, Secretary, and Dr. T. J. Watkins, Editor. The retiring president, Dr. Fernand Henrotin, delivered an interesting annual address, after which the Society adjourned to the annual banquet. Dr. John B. Hamilton, J. B. Murphy, Health Commissioner Arthur Reynolds, Alex. H. Ferguson and others were guests of the Society.

